# Unilateral vision loss as the initial symptom of chronic myeloid leukemia with bilateral retinal infiltration: a case report

**DOI:** 10.1186/s12886-026-04995-3

**Published:** 2026-06-19

**Authors:** Wei Chen, Bo Qin

**Affiliations:** 1 Department of Ophthalmology, The University of HongKong-Shenzhen Hospital, Shenzhen, Guangdong 518053 PR China; 2 Shenzhen Aier Eye Hospital,Aier Eye Hospital, Jinan University, No.2048, Huaqiang South Road, Futian District, Shenzhen, Guangdong 518032 PR China; 3 Aier Academy of Ophthalmology, Central South University, No.188, Furong South Road, Tianxin District, Changsha, Hunan 410004 PR China

**Keywords:** Ophthalmic manifestations, Chronic myeloid leukemia, Philadelphia chromosome

## Abstract

**Background:**

Ophthalmic manifestations as the initial presentation of chronic myeloid leukemia (CML) are rare.

**Case presentation:**

This case describes a 38-year-old man who presented with unilateral severe vision loss alongside bilateral ocular findings, including intraretinal hemorrhages, vascular tortuosity, cotton wool spots, and optic disc edema. Ophthalmic imaging, particularly optical coherence tomography (OCT), revealed bilateral retinal involvement, which was more severe in the left eye despite worse vision in the right eye. Systemic evaluation revealed critical hyperleukocytosis, splenomegaly, and Philadelphia chromosome positivity, confirming CML. The subsequent MRI during remission showed no abnormality. Treatment with hydroxyurea and nilotinib rapidly normalized white blood cell counts and reached major molecular response (MMR) along with complete visual recovery and normal retina and optic nerve.

**Conclusions:**

This case highlights the importance of recognizing ocular signs as potential indicators of life-threatening systemic diseases such as CML, warranting prompt diagnosis and intervention. Ocular signs not only help to confirm the primary diagnosis, but also may serve as a useful adjunctive marker for monitoring treatment response.

## Background

Ophthalmic manifestations are considered uncommon presentations of chronic myeloid leukemia (CML), with only case reports or small case series. A literature review conducted up to 2022 identified thirty-eight published articles describing ocular manifestations in patients with CML [1]. These ocular complications include tortuosity of veins, intraretinal or preretinal hemorrhages, and cotton-wool spots [2], which can be owing to leukemic infiltration and leukemic retinopathy caused by other factors such as anemia, thrombocytopenia, leukocytosis, and hyperviscosity syndrome [3]. Other rare ophthalmic manifestations reported in recent years include rhegmatogenous retinal detachment, proliferative retinopathy, leopard-spot retinopathy, and optic neuropathy [4–7]. Ophthalmic manifestations, the initial and sole presentations of CML in patients, are very rare [8]. Advances in multimodal ophthalmic imaging have facilitated timely diagnosis and detection of early changes in patients [9]. Patients with CML, if diagnosed in a timely manner and treated appropriately, reportedly have a survival rate of 90% [10]. Leukemic retinopathy has historically been considered a poor prognostic indicator [11]. Therefore, early diagnosis is crucial. Here, we report a rare case of bilateral ocular involvement with severe unilateral vision loss as the initial manifestation of CML.

## Case presentation

**Fig. 1:**
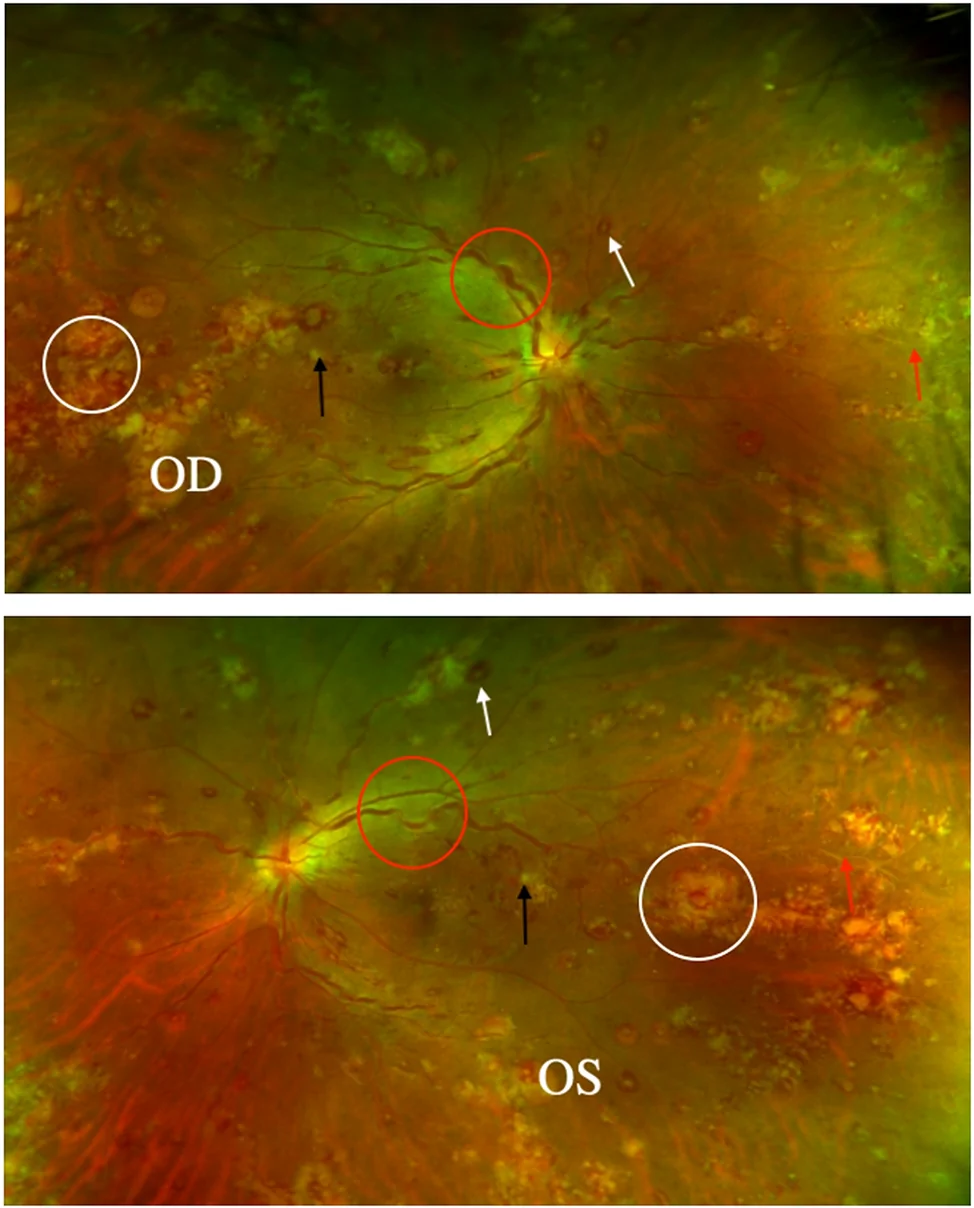
Funduscopic examination revealed bilateral clear vitreous, multiple Roth spots (white arrows), diffuse retinal infiltrates (white circles), vascular tortuosity (red circles), peripheral vascular sheathing (red arrows) and optic disc edema. Both macula presented with cotton-wool spots (black arrows)

**Fig. 2:**
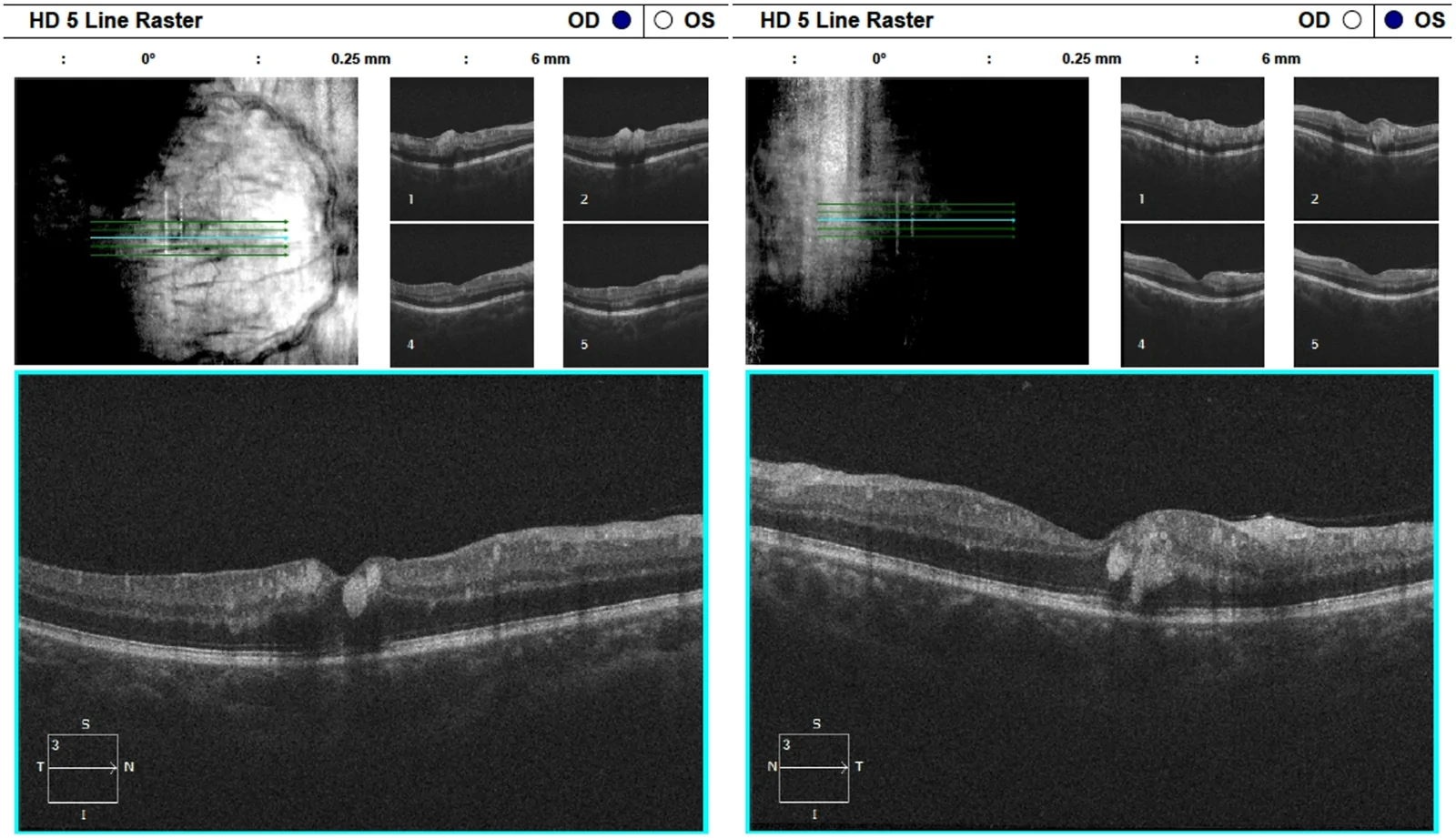
OCT on horizontal scans through the fovea revealed multiple hyperreflective foci throughout the inner nuclear, inner plexiform, outer nuclear, and outer plexiform layers, hyperreflective thickening of the retinal nerve fiber layer. The left eye showed more severe disorganization than the right eye

A 38-year-old man presented with a one-week history of severe visual loss in the right eye. He reported sleep disturbances for approximately one year and had been taking zolpidem tartrate prescribed by the neurology department. There was no specific family history or other systemic diseases, such as tuberculosis (TB), trauma, hypertension, or diabetes mellitus (DM). His best-corrected visual acuity (BCVA) was reduced to 0.1 (+ 0.50ds/-0.50dc*8) in the right eye and 0.9 (-0.75ds/-0.50dc*133) in the left eye. Intraocular pressure and anterior segment examination results were normal in both eyes. Funduscopic examination (Fig. 1) revealed bilateral clear vitreous, multiple intraretinal hemorrhages with white centers (Roth spots), diffuse retinal infiltrates, vascular tortuosity, peripheral vascular sheathing and optic disc edema. Cotton-wool spots were observed at the macula bilaterally. Optical coherence tomography (OCT) (Fig. 2) of horizontal scanned through the fovea revealed multiple hyperreflective foci throughout the inner nuclear, inner plexiform, outer nuclear, and outer plexiform layers. The volume of hyperreflective foci in the fovea of the left eye’s macula was larger than that in the right eye, and the number of foci was also greater. The disorganization of the inner retinal layers in left eye’s macula was worse than that of right eye, which was inconsistent with the patient’s visual acuity.

On the basis of the history and ophthalmological findings, systemic diseases such as tuberculosis, syphilis, leukemia, and multiple myeloma were considered. The full blood count results (Table 1) revealed critically elevated white blood cell (WBC) counts of 531.62*10^9^/L. Other vital serological items (Table 2) were positive for HBsAg, anti-HBc, and HBeAg and negative for HIV and TP. A chest X-ray was unremarkable. The patient was immediately transferred to the hematology department.

**Table 1:** Routine blood test results

Blood routine test report				
Item	Result	Flag	Unit	Reference interval
White Blood Cell Count (WBC)	531.62	↑↑↑	10^9/L	3.89–9.93
Hemoglobin (Hb)	72	↓	g/L	133–171
Platelet Count (PLT)	182	↓	10^9/L	162–341
Red Blood Cell Count (RBC)	2.13	↓	10^12/L	4.46–5.71
Hematocrit (Hct)	20.4	↓	%	39.5–48.8
Mean Corpuscular Volume (MCV)	95.8	↑	fL	82.0-95.5
Mean Corpuscular Hemoglobin (MCH)	33.8	↑	pg	27.0-32.4
Mean Corpuscular Hemoglobin Concentration (MCHC)	353		g/L	326–357
Red Cell Distribution Width (RDW-CV)	21.8	↑	%	11.2–13.9
Film Review Performed?	Yes			
Neutrophils % (Neut%)	34	↓	%	44.0–72.0
Lymphocytes % (Lym%)	3	↓	%	20.0–45.0
Monocytes % (Mon%)	2		%	2.0–10.0
Eosinophils % (Eos%)	4		%	1.0–6.0
Basophils % (Baso%)	3	↑	%	0.0–1.0
Neutrophils Absolute (Neut#)	180.75	↑	10^9/L	2.01–7.42
Lymphocytes Absolute (Lym#)	15.95	↑	10^9/L	1.06–3.61
Monocytes Absolute (Mon#)	10.63	↑	10^9/L	0.18–0.65
Eosinophils Absolute (Eos#)	21.26	↑	10^9/L	0.02–0.45
Basophils Absolute (Baso#)	15.95	↑	10^9/L	0.01–0.07

**Table 2:** Infectious disease serology test report

Test item	Result	Unit	Reference interval
Hepatitis B Surface Antigen (HBsAg)	2.23	IU/mL	< 0.05
Hepatitis B e-Antigen (HBeAg)	Negative		Negative (NEG)
Hepatitis B e-Antibody (Anti-HBe)	Positvie		Negative (NEG)
Hepatitis B Core Antibody (Anti-HBc)	Positvie		Negative (NEG)
HIV Antigen/Antibody (HIV Ag/Ab Combo)	Negative		Negative (NEG)
Hepatitis C Virus Antibody (Anti-HCV)	Non-Reactive		Non-Reactive
*Treponema pallidum* Antibody (Syphilis TP)	Non-Reactive		Non-Reactive

In the hematology department, no additional history was obtained. Physical examination was unremarkable except for splenomegaly. The physical examination showed spleen size below costal margin about 6 cm, which was exactly recorded by the computed tomography (CT) scan and B scan ultrasonography: splenomegaly (thickness: 67 mm, length: 208 mm), with the inferior pole extending 54 mm below the umbilical level. Emergency bone marrow aspiration and biopsy were performed.

**Fig. 3:**
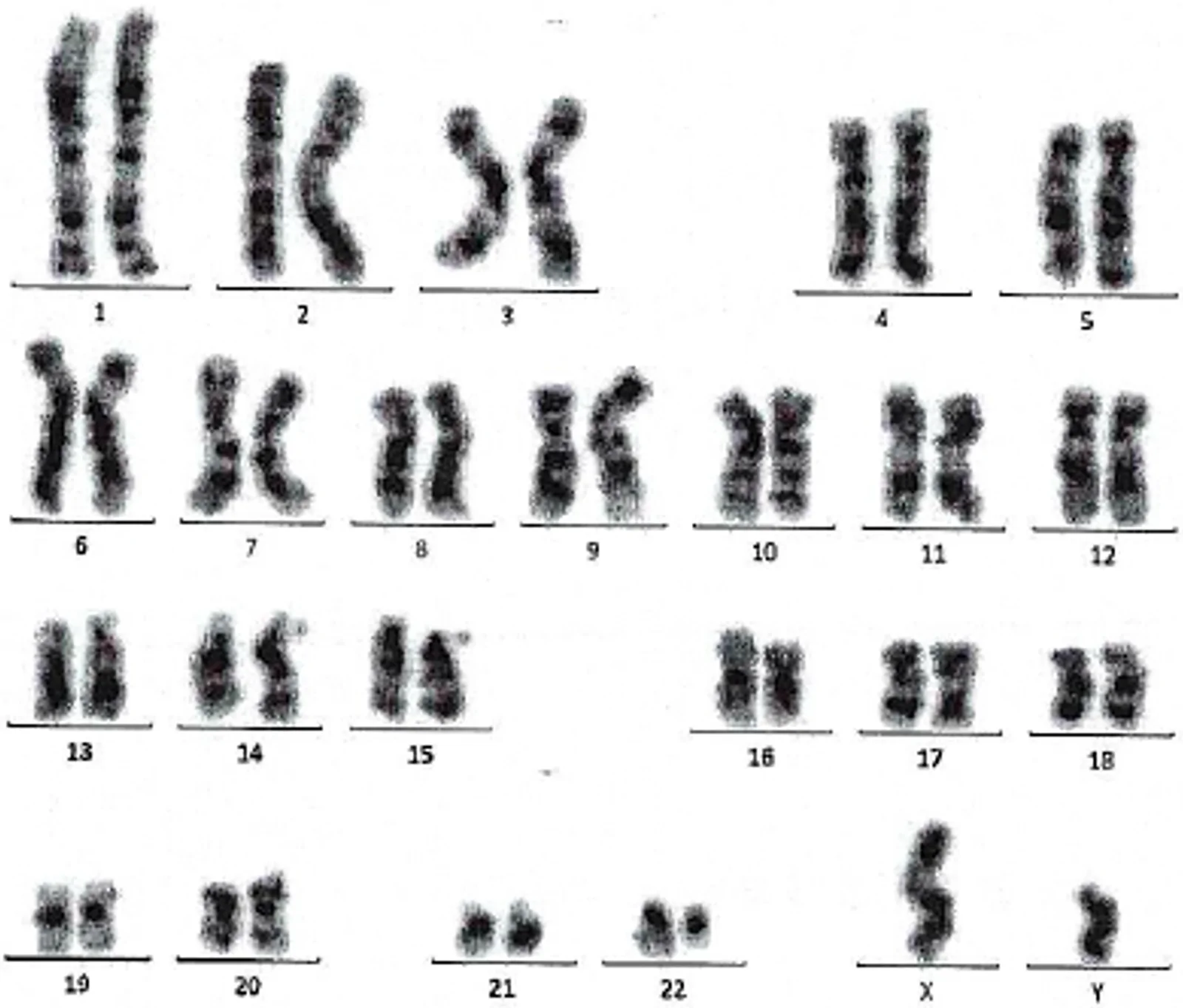
Karyotype analysis result (ISCN): Karyotype: 46, XY, t(9;22)(q34;q11.2) [12]/46, idem, add(5)(q31). The karyotype results in this report are described according to ISCN 2020. Twenty metaphase cells were analyzed. A reciprocal translocation between the long arms of one chromosome 9 and one chromosome 22 was observed in all the cells. This is an acquired genetic variation; t(9;22) can be seen in CML, ALL, and AML. An unknown segment attached to the long arm of chromosome 5 was also observed in some cells

The peripheral blood smear (Table 3) revealed a markedly increased white blood cell count and a relative decrease in the lymphocyte percentage. The platelet and monocyte counts were within normal limits. Peripheral blood smears and bone marrow (Table 4) findings were compatible with CML. The BCR/ABL1 fusion gene (Table 5), present in more than 95% of CML cases and 10–20% of acute lymphoblastic leukemia cases, was detected. The karyotype result in this report was described according to ISCN 2020: karyotype: 46, XY, t(9;22)(q34;q11.2) [12]/46, idem, add (5)(q31) (Fig. 3). The Philadelphia (Ph) chromosome, formed by reciprocal translocation between 9q34 and 22q11.2, was a specific marker chromosome for CML and is an acquired genetic variation.

**Table 3:** Peripheral blood examination findings

Parameter	Finding	Unit
Hemoglobin (Hb)	67	g/L
White Blood Cell Count (WBC)	413.69	10^9/L
Platelet Count (Plt)	175	10^9/L
Blood Smear		
1. White Blood Cells	Markedly increased white blood cell count.	
2. Differential Count (200 cells)	Classification is predominantly granulocytic:	
	- Myeloblasts: 0.6%	
	- Promyelocytes: 4.0%	
	- Neutrophilic myelocytes: 18.0%	
	- Neutrophilic metamyelocytes: 23.0%	
	- Neutrophil band forms & segmented neutrophils: 46.0%	
	- Eosinophils: 3.0%	
	- Basophils: 3.0%	
	- Lymphocytes: 3.0%	
	- Monocytes: 1.0%	
	No significant morphological abnormalities are noted.	
3. Red Blood Cells	Mild anisocytosis. No significant morphological abnormalities. 1 nucleated red blood cell is seen per 200 white blood cells.	
4. Lymphocytes & Monocytes	Relative decrease in the lymphocyte percentage. Monocyte count shows no significant increase or decrease.	
5. Platelets	Platelet number shows no significant increase or decrease. Size, morphology, and distribution appear normal without significant abnormality.	

**Table 4:** Bone marrow examination findings

Parameter	Finding
Bone marrow aspirate	
1. Specimen Quality	Adequate specimen procurement, smear preparation, and staining.
2. Marrow Particles	Marrow particle area > 90%, predominantly composed of hematopoietic cells.
3. Cellularity & M:E Ratio	Markedly hypercellular. Myeloid to erythroid (M: E) ratio is significantly increased.
	- Granulocytic series (G): 30.2%
	- Erythroid series (E): 4.6%
	- M: E Ratio: 19.6:1
4. Granulopoiesis (500 cells)	Markedly hyperplastic with an increased number of granulocytes at all stages:
	- Myeloblasts: 0.4%
	- Promyelocytes: 3.6%
	- Neutrophilic myelocytes: 20.0%
	- Neutrophilic metamyelocytes: 24.0%
	- Neutrophil band & segmented forms: 36.0%
	- Eosinophils: 6.8%
	- Basophils: 2.0%
	No significant morphological abnormalities noted.
5. Erythropoiesis	Relatively decreased in proportion, primarily consisting of polychromatic and orthochromatic normoblasts. Morphology is unremarkable. Mature red blood cell morphology is consistent with peripheral blood findings.
6. Lymphocytes & Monocytes	Relative decrease in lymphocyte proportion. Monocytes show no significant increase or decrease.
7. Megakaryopoiesis	Increased in number with adequate platelet production. Classification shows predominantly granular megakaryocytes and platelet-producing megakaryocytes. Megakaryocytes are mainly small, hypolobated (“dwarf megakaryocytes”). Small platelet clusters are easily found.
8. Other	No blood parasites identified.
Cytochemical Stains	
Iron Stain	- Extracellular iron: 1+
	- Intracellular iron (siderotic granules): 36%
	- No ringed sideroblasts identified.
Neutrophil Alkaline Phosphatase (NAP)	- Positive rate: 0%
	- Score: 0
Pathologic Diagnosis	Peripheral blood smear and bone marrow findings are compatible with Chronic Myeloid Leukemia (CML). To await trephine biopsy, Ph chromosome analysis, and BCR-ABL1 fusion gene testing for further confirmation. Please correlate with clinical context.

**Table 5:** BCR/ABL1 fusion gene test

Probe name	Result	International system for human cytogenomic nomenclature (ISCN2020)
LBP BCR/ABL fusion gene	Positive	nucish (ABL1, BCR) ×2 (ABL1 con BCR×1) [189/200]
Signal Pattern Results:	1. The BCR/ABL1 fusion gene was detected.	
	2. 200 cells were analyzed. The respective signal patterns were as follows: 1G1R1F 94.5%, 2G2R 3.0%, 2G1F 2.5%.	
Interpretation:	1. The green fluorescent signal marks the BCR (22q11.2) probe, and the red fluorescent signal marks the ABL1 (9q34) probe. The BCR/ABL1 fusion gene is displayed as a yellow or red‒green (F) signal. The normal signal pattern is 2G2R (Note: G represents green signals, R represents red signals, F represents fusion signals).	
	2. The Philadelphia (Ph) chromosome, formed by the reciprocal translocation between 9q34 and 22q11.2, is a specific marker chromosome for CML. The BCR/ABL1 fusion gene is present in over 95% of CML patients and 10%-20% of acute lymphoblastic leukemia (ALL) cases. It has subsequently been found in AML and some other hematological disorders.	
	3. Present in 20–25% of CML patients in the blast crisis phase, it serves as a marker of disease progression and may be an indicator of Gleevec (imatinib) resistance. The presence of the BCR/ABL1 fusion gene in ALL suggests a poor prognosis.	
	4. FISH technology can be used for the initial diagnosis of patients, identification of variant Ph (complex or cryptic) translocations, monitoring of treatment efficacy, and prognostic assessment.	

On the basis of these findings, the patient was diagnosed with CML and blood hyperviscosity, indicating a critical condition. Treatment was initiated with 0.5 g of hydroxyurea three times daily, followed by treatment with the tyrosine kinase inhibitor (TKI) -Nilotinib.

**Fig. 4:**
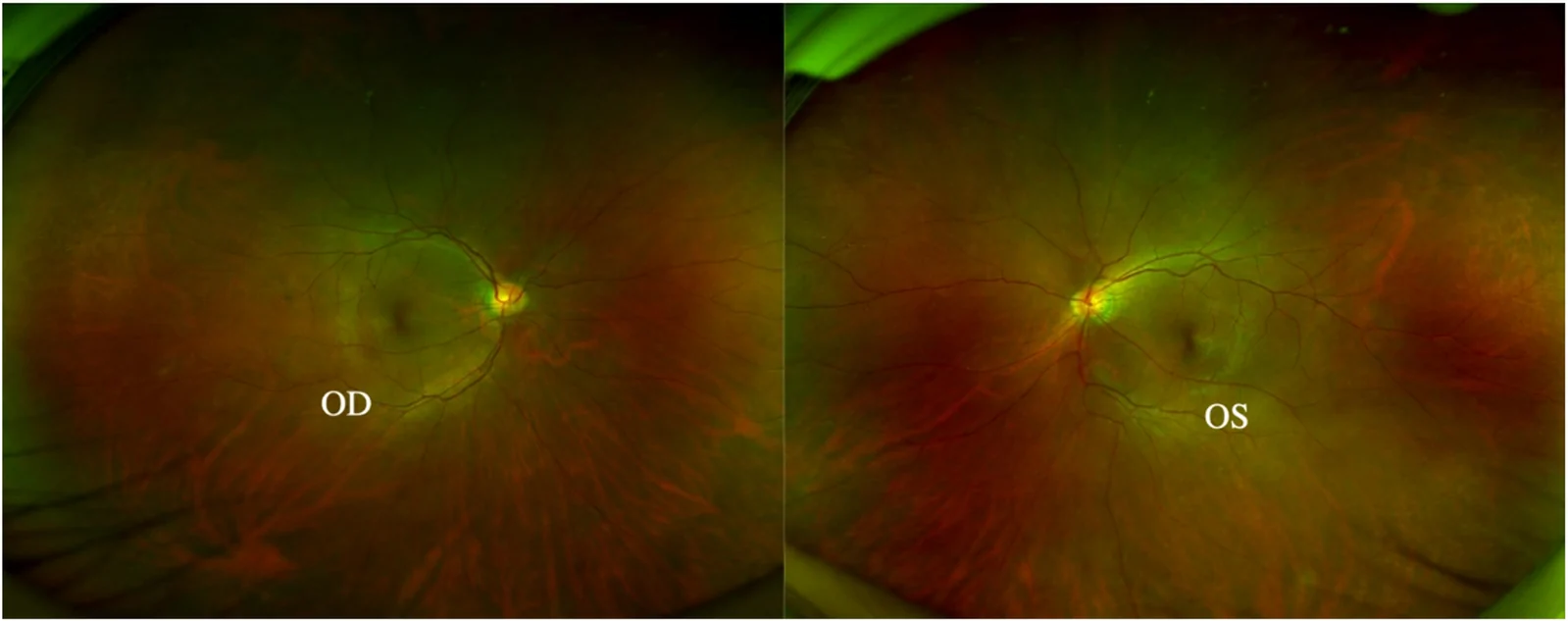
Follow-up fundus photographs showed normal retina without optic nerve edema in both of eyes

**Fig. 5:**
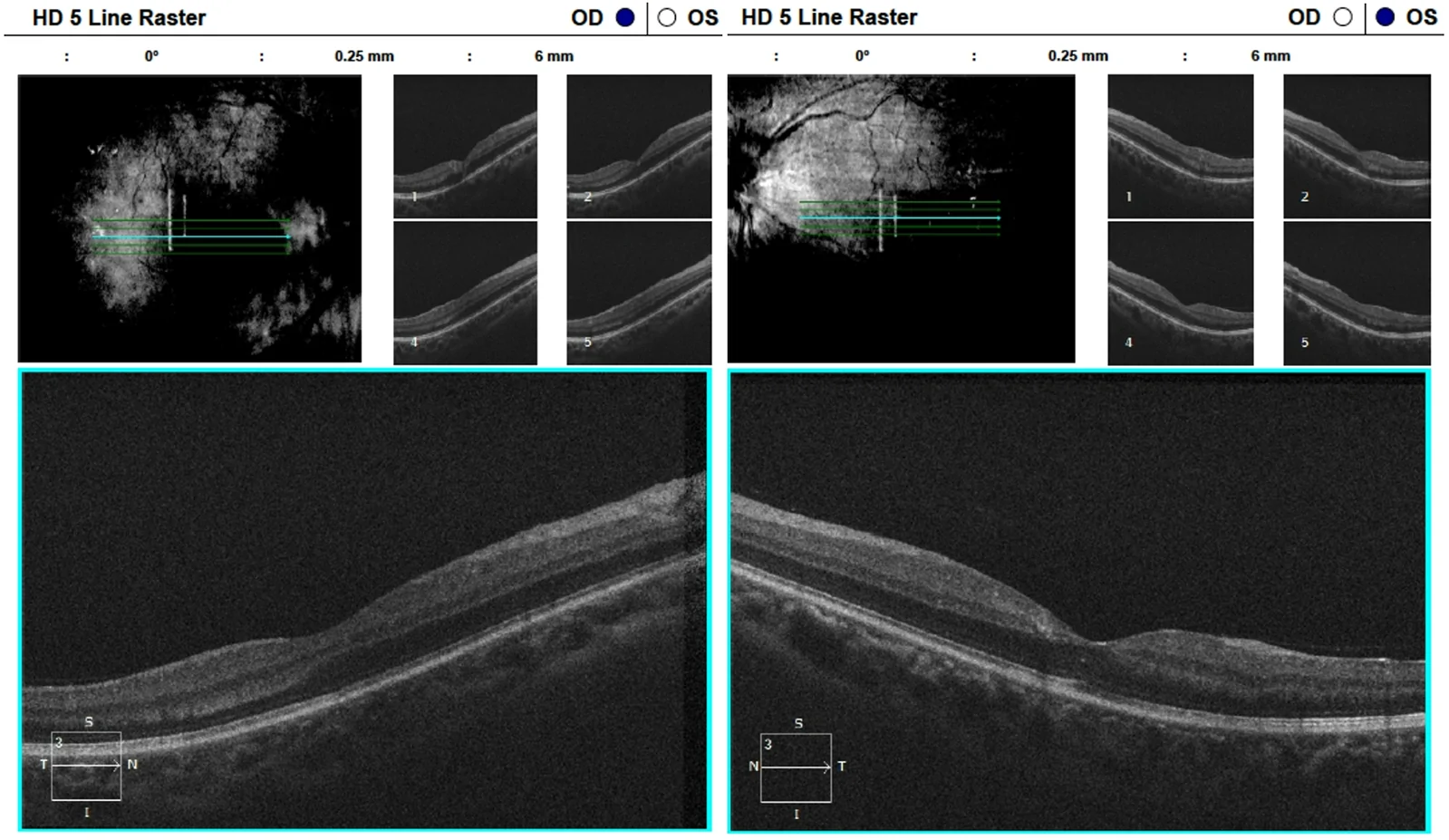
OCT on horizontal scans through the fovea revealed no significant multiple hyperintense reflections throughout the inner layers

**Fig. 6:**
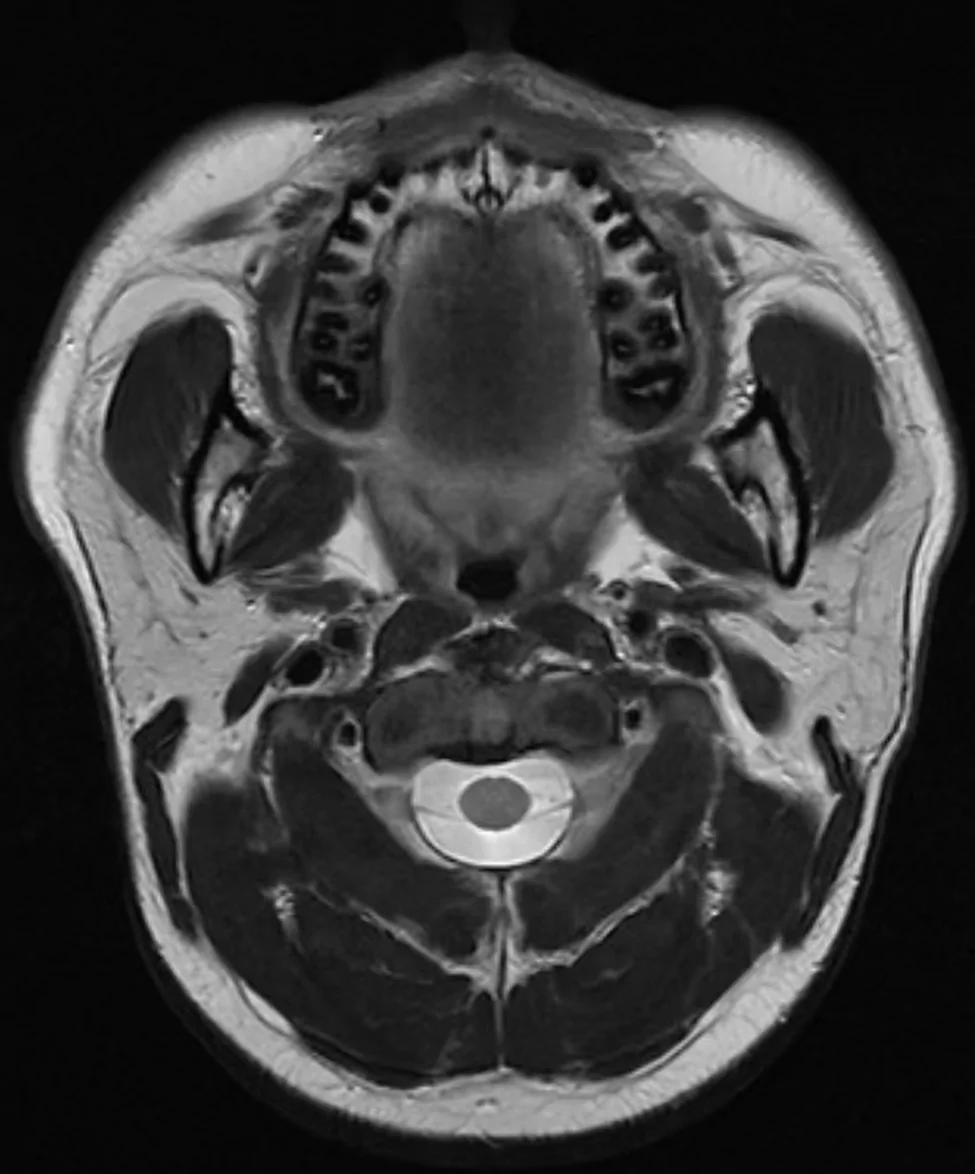
MRI images obtained during the patient’s remission period revealed no significant abnormalities in brain and orbital

**Fig. 7:**
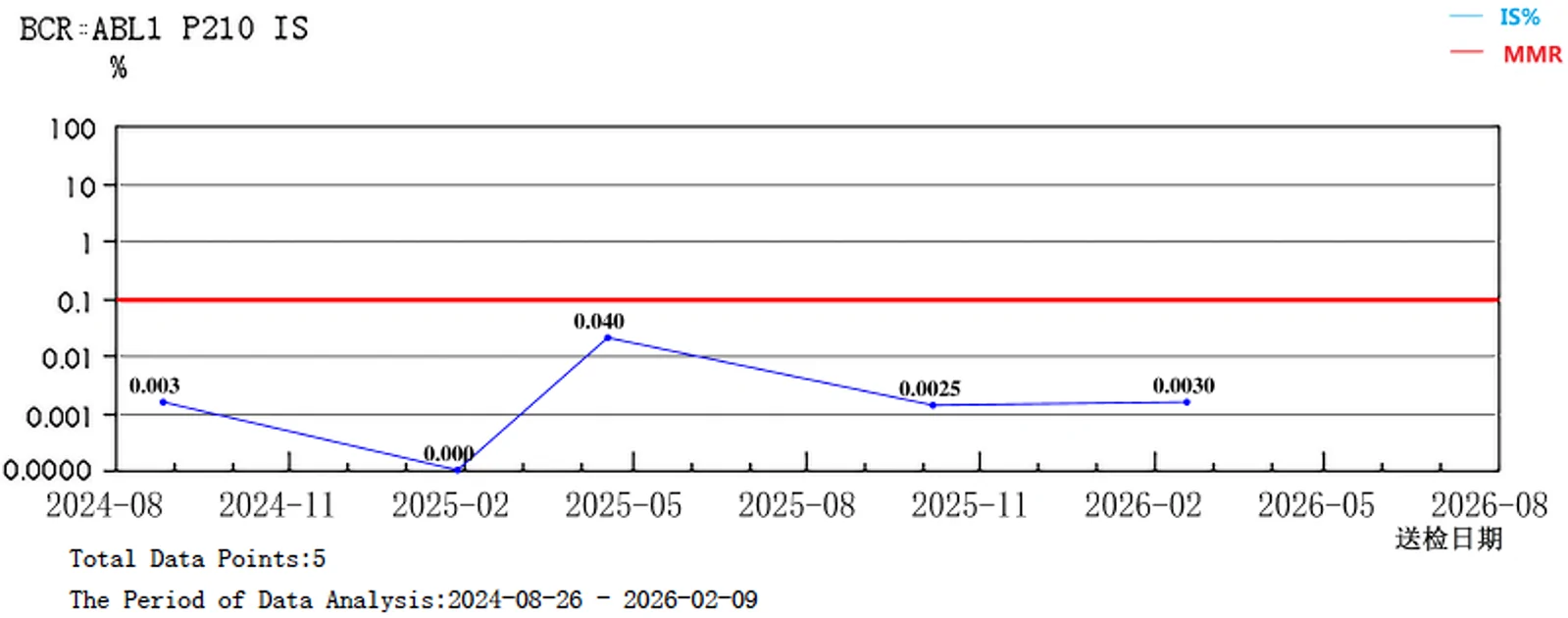
Dynamic monitoring map of BCR-ABL1 (p210) Fusion Gene quantitative test from August 26th^th^ 2024 to February 09^th^ 2026

After three weeks of treatment, the patient’s WBC count decreased to 3.37 × 10⁹/L. Follow-up fundus photographs showed normal retina without optic nerve edema (Fig. 4) and OCT revealed no significant hyperreflective foci in the inner layers (Fig. 5) in both eyes. MRI images (Fig. 6) obtained during the patient’s remission period revealed no significant abnormalities in brain and orbital. Hematological response and major molecular response (MMR) were monitored till February 09th 2026 during patient’s treatment (Table 6; Fig. 7), which represented he was in the stable stage. Patient’s uncorrected visual acuity was 1.0 both eyes.

**Table 6:** BCR-ABL1 (p210) fusion gene quantitative test report

Submission date	BCR::ABL1 copies	ABL1 copies	BCR::ABL1/ABL1 (%)	BCR::ABL1 (IS) (%)	MR value	Major molecular response
2026/2/9	11	243,902	0.0045	0.0030	4.52	Yes
2025/9/30	21	564,876	0.0037	0.0025	4.6	Yes
2025/4/14	66	109,976	0.060	0.040		Yes
2025/1/26	0	172,866	0.000	0.000		Yes
2024/8/26	10	214,758	0.005	0.003		Yes

PS: All specimen type of these test were peripheral blood

## Discussion and conclusions

Typical leukemic retinopathy features, such as cotton wool spots and Roth spots, are regarded as nerve fiber layer ischemia and platelet-fibrin aggregation, respectively [13]. The nature of multiple intra-retinal hyper-reflective foci in OCT was corresponded to intra-retinal hemorrhages, and outer retinal hyper-reflective foci in area corresponding to retinal infiltrate [14]. Other significant features include dilated tortuous vessels, optic disc edema and macular hemorrhage [15]. Several hematologic diseases can mimic leukemic retinopathy and present similar fundus signs, including anemic retinopathy, sickle-cell retinopathy, and lymphoma-associated retinopathy [16].

Ocular manifestations as the initial symptoms in only 5%-10% of patients diagnosed with CML [7]. Ophthalmic involvements are more common in acute than in chronic leukemias [17]. Patients with CML had more frequent posterior pole involvement, requiring close follow-up for possible sight-threatening [18]. Although CML patients with ocular manifestations have been reported to have lower 5-year survival rates than those without ocular manifestations do, prompt treatment results in good recovery [17, 19]. However, one study by Gulfidan et al. reported no significant difference in fatality between CML patients with and without ocular involvement [12].

Since central nervous system (CNS) involvement of CML is rare [20, 21], lumbar puncture and MRI was not conducted onset in this case. In our case, funduscopic examination revealed similar involvements in retina and optic nerve. Meanwhile OCT revealed more pronounced structural involvement in the left eye than in the right eye, yet the right eye had greater visual impairment. This apparent discrepancy could be attributed to the OCT topography of the lesions: foveal involvement in the right eye versus a juxtafoveal distribution in the left eye. Through medical treatment, WBC normalization and BCR-ABL1 (p210) Fusion Gene MMR were accompanied by visual improvement and normal ocular image presentation in both eyes. The reason of the vision loss remains uncertain, but several mechanisms may be considered to attribute to this uncommon presentation. One possible mechanism could be leukemic cells infiltration of the optic nerves [22]. Yassin et al.’s review revealed that, in several cases, ophthalmological involvement presented with both eyes but decreased vision in one eye as the onset symptom. In their survey, the optic nerve infiltration of a patient who experienced optic nerve edema was tested via MRI enhancement [1]. However without timely MRI could not prove this implication. Another potential mechanism could be hyperviscosity syndrome. In previous studies, haemorrhagic manifestations were regarded as a consequence of secondary anaemia, thrombocytopenia and hyperviscosity syndrome [18]. Hyperviscosity syndrome-related retinopathy has been reported as intraretinal hemorrhages, vascular dilation and tortuosity, and optic disc edema [23]. In our case, WBC count more than 500*10^9^/L, which may have led to reduced blood flow and high risk of thrombosis, particularly affecting the microcirculation resulting in ischemia and optic nerve edema. A pilot cross-sectional study to evaluate visual evoked potential (VEP) in 20 newly CML cases demonstrated that prolonged latency in PVEP suggested demyelination of the optic nerve due to ischemia caused by the slowing of blood as a consequence of hyperviscosity. Hence, they inferred that VEP has paramount importance in the early identification of ophthalmic manifestations [24].

The present case report has several limitations. Firstly, we did not perform MRI or lumbar puncture timely at the onset of the patient’s symptoms to determine whether the optic disc edema was caused by CNS infiltration or other intracranial diseases. Although previous literature suggested that CNS involvement in CML was rare, a subsequent MRI obtained three years after onset, during the patient’s stable remission, which could help exclude other intracranial causes of optic disc edema at that time, it can not rule out whether optic nerve infiltration was present at the initial presentation. Secondly, we lacked clarification of the patient’s prior neurological evaluation. In our case, the patient camplained of sleep disturbances for approximately one year. Although previous studies revealed that sleep disturbance was indeed a common symptom in CML patients, we still can not clarify if it was CML itself or other, such as hyperviscosity, mediated this symptom [25, 26]. Finally, the exact cause of the patient’s severe unilateral vision loss remained undetected. Although we speculated the visual impairment discrepancy could be explained by OCT topography of lesions, we can not confirm it without fundus fluorescein angiography (FFA) nor OCT angiography (OCT-A) to assess the ischemia of optic nerve and peripheral retina. FFA was not performed at presentation because the patient was in a life-threatening state, and it was uncertain whether FFA would have caused additional harm. More comprehensive investigations, such as the optic disc OCT to measure the degree of optic nerve edema, OCT-A to assess the degree of macular and optic nerve ischemia and visual field to assess the specific location of visual function impairment, are needed in the future to further elucidate the etiology of this condition and assess the impairment of visual functions.

Unilateral vision loss with bilateral ocular involvement as the initial presentation of CML is exceedingly rare. Since CML-related ocular manifestations can resemble those of other infectious or vascular retinopathies, ophthalmologists should be aware of this potentially life-threatening etiology to ensure prompt diagnosis and treatment. Ocular signs not only help to confirm the primary diagnosis, but also may serve as a useful adjunctive marker for monitoring treatment response. Deeper investigations related to CML and vision damage, as well as CML progression and ophthalmological multimodal images, should be considered in future studies.

## Data Availability

The data supporting this study’s findings are available from the corresponding author upon reasonable request.

## Abbreviations

CML: Chronic myeloid leukemia
TB: Tuberculosis
DM: Diabetes mellitus
OCT: Optical coherence tomography
WBC: White blood cell
CT: Computed tomography
TKI: Tyrosine kinase inhibitor
BCVA: Best-corrected visual acuity
VEP: Visual evoked potential
MMR: Major molecular response
CNS: Central nervous system
OCT-A OCT: Angiography
